# The effect of a broad spectrum sunscreen with visible light coverage on facial photoaging: A pilot study

**DOI:** 10.1111/jocd.16492

**Published:** 2024-07-23

**Authors:** David F. Godoy, Francisco E. Villanueva, Andrea A. Cortés, Viviana Zemelman

**Affiliations:** ^1^ Department of Dermatology, Faculty of Medicine Universidad de Chile Santiago Chile; ^2^ Arturo López Pérez Foundation (FALP) Cancer Institute Santiago Chile; ^3^ Dermatology Service Hospital Clínico Universidad de Chile Santiago Chile

To the Editor, skin aging involves clinical, histological, and physiological changes due to intrinsic factors (chronological aging) and extrinsic factors such as the exposome.^1^ Photoaging manifests as wrinkles, lentigines, telangiectasias, and loss of elasticity. UV radiation induces damage to connective tissue through reactive oxygen species (ROS).^2^

Visible light, especially blue light, also contributes to photoaging by generating ROS.^3^ Although most sunscreens defend against UVB and short UVA radiation, few FDA‐approved sunscreens provide coverage against long UVA radiation, and none against visible light.^4^ Limited data exist on sunscreen efficacy in preventing photoaging.^5^ In a randomized clinical study, it was observed that regular daily use of a photoprotector reduces photoaging assessed on the back of the hand.^6^

The objective of this study was to determine whether the use of a new sunscreen with visible light coverage reduces the clinical parameters associated with skin photoaging in a cohort of healthcare workers.

A non‐randomized interventional clinical study was carried out on 50 subjects over 18 years of age at a university hospital. The subjects were instructed to use ANTHELIOS UV MUNE 400® (LaRoche Posey, France) three times a day for 8 weeks. Facial and dermoscopic images were analyzed at the start of the study (Day 1) and after 8 weeks using sunscreen (Day 56) with the VISIA® Skin Analysis System and the DPAS dermoscopy scale to assess skin aging.

Of the 50 participants, 39 completed the two assessments, ranging in age from 22 to 66 years and averaging 45.3 years (*SD* ±10.9). The subjects who completed the study showed a clear predominance of the female sex (87.2%) (Table 1).

**TABLE 1:** Demographic characteristics and facial skin analysis of healthcare workers.

Demographic variables	
Cases (*n*)	39
Mean age (*SD*)	45.3 (10.9)
Gender, female [*n* (%)]	34 (87.18)
Gender, male [*n* (%)]	5 (12.82)

Facial skin analysis	Day 1 (percentile)	Day 56 (percentile)	Difference (CI 95%)	*p‐*Value
VISIA®				
Wrinkles	36.2	40.1	−3.9 (−12.2; 4.4)	0.34
UV spots	79.7	89.5	−9.7 (−15.3; −4.0)	0.0013
Brown spots	33.8	36.5	−2.6 (−9.5; 4.2)	0.4
Spots	33.3	39.87	−6.57 (−14.5; 1.3)	0.0516
Texture	30.94	27.71	3.23 (−1.2; 7.7)	0.07595
Pores	33.94	35.05	−1.11 (−6.3; 4.1)	0.3359
Porphyrins	70.25	83.94	−13.69 (−0.2; −0.06)	0.0003
Overall score	46.4	51.2	−4.7 (−8.2; −1.2)	0.0085
DPAS				
DPAS score	9.97	8.76	1.2 (0.53; 1.87)	0.0009

*Note*: Means and proportions were compared with Student's *t*‐test, Mann–Whitney *U*‐test, Chi‐square and Fisher's exact test, as appropriate, and Person's or Spearman's correlation coefficient was used depending on the relationship. The significance level was *p*‐value < 0.05.

Abbreviations: CI, confidence interval; DPAS, dermoscopic photoaging scale; *SD*, standard deviation.

When comparing the overall result of the eight parameters provided by the VISIA® software, it was noticed that before the treatment, participants were in the 46th percentile, while in the second evaluation, carried out on Day 56 of the study, an improvement of 4.77 points was recorded, reaching the 51st percentile (*p*‐value 0.0085). When analyzing the individual parameters associated with photoaging, there was a 10‐point shift in UV spots toward percentiles with fewer UV spots (*p*‐value 0.0013). For wrinkles and brown spots, there was an improvement in percentile but this difference was not statistically significant (Table 1).

In addition, skin aging was assessed by another method using the DPAS dermoscopy scale before and after wearing sunscreen showing a significant decrease in the score of 1.2 points (*p*‐value 0.0009) (Table 1), in line with what was observed using the VISIA® software.

The present study determined the effect of a sunscreen with visible light coverage on a cohort of adult subjects after a short‐term use showing a significant improvement in the parameters of facial aging analyzed by two different methodologies: the 8‐parameter analysis of the VISIA® software and the DPAS dermoscopy scale. This result is novel because of the limited number of clinical studies that have determined in vivo the effect of sunscreens in the treatment or prevention of facial photoaging,^5^ and this study is one of the first to test a sunscreen with visible light coverage.

Some of the limitations of the present study include the relatively small number of participants, being a single‐center study, the short follow‐up period, which prevents us from observing a long‐term preventive effect of photoprotection use, and the lack of a control group. Future studies should compare sunscreens with and without visible light coverage to determine their long‐term effects on facial photoaging.

In conclusion, the use of sunscreen with visible light coverage proved to have a positive effect on improving photoaging.

## FUNDING INFORMATION

The Dermatology Service of Universidad de Chile received funds from L'Oréal Chile S.A. to finance this study.

## CONFLICT OF INTEREST STATEMENT

None to declare.

## ETHICS STATEMENT

The study protocol was approved by the Ethics Committee of the Universidad de Chile Clinical Hospital, approval record N°030, May 18, 2022. All study participants signed a written informed consent prior to the start of the study.

## Data Availability

The data underlying this article will be shared on reasonable request to the corresponding author.
